# Grand challenge in biosafety and biosecurity

**DOI:** 10.3389/fbioe.2024.1538723

**Published:** 2025-01-07

**Authors:** Stephen A. Morse

**Affiliations:** IHRC, Inc, Atlanta, GA, United States

**Keywords:** high-containment laboratories, dual-use-research, biorisk management, synthetic biology, nucleic acid synthesis

## Introduction

The Biosafety and Biosecurity Section of Frontiers in Bioengineering and Biotechnology began in 2014 to serve as the forum to address many of the biosafety and biosecurity challenges posed by bioengineering and biotechnology. Although there are many areas to highlight in a Grand Challenges article, captured herein are a few topics that are of current and future concern to the field.

## Frontier 1: expansion of high-containment laboratories

Anticipating the biosafety and biosecurity challenges to the global expansion of high containment laboratories was subject of an international workshop held more than a decade ago (National Academy of Sciences and National Research Council, 2012). The meeting covered: 1) Technological options to meet diagnostic and research needs; 2) Laboratory construction and commissioning; 3) Operational maintenance to provide sustainable capabilities, safety, and security; and 4) Measures for encouraging a culture of responsible conduct. Since that meeting, new, emerging and re-emerging infectious diseases such as SARS-CoV, MERS, Ebola, Zika, H5N1 avian influenza, SARS-CoV-2, and monkeypox have resulted in a further increase of high containment laboratories (Lentzos and Koblentz, 2023). The proliferation of the number of BSL-4 and BSL-3+ facilities have elevated risks as well as demand for improved training, standards, and oversight (Morrison and Simoneau, 2023).

Many of the high containment labs are in or planned for urban centers where an accidental or intentional release could be problematic (Klotz and Sylvester, 2014). In fact, several outbreaks have been linked or suggested to be the result of pathogens escaping from a laboratory: influenza H1N1 in 1977 (Palese, 2004), SARS-CoV in 2004 (Manheim and Lewis, 2022), and possibly SARS-CoV-2 (Gostin and Gronvall, 2023). It is also particularly concerning that several countries that have built or plan to build BSL-4 and BSL-3+ laboratories scored low on the metrics used to assess their biosafety and biosecurity capabilities (Lentzos and Koblentz, 2023).

From a global perspective, biorisk management has been proposed to address both biosafety and biosecurity in laboratories (Salerno and Gaudioso, 2015; Rodgers et al., 2021). Because biorisk management is oriented around performance, a laboratory in a low-resource country can implement it as effectively as one in a developed country. To help inexperienced laboratories as well as those with experience, the International Organization for Standardization (ISO) released ISO 35001:2019, a standard on biorisk management for laboratories that work with dangerous pathogens (https://www.iso.org/standard/71293.html). To further address these concerns, the World Health Organization (WHO) developed the “*Global Guidance Framework for the Responsible Use of the Life Sciences: Mitigating Biorisks and Governing Dual-Use Research*” (WHO, 2022). This biorisk management framework encompasses three core pillars: laboratory biosafety, laboratory biosecurity and the oversight of dual-use research (WHO, 2022).

## Frontier 2: dual-use-research of concern

Dual-Use Research of Concern (DURC) is defined as “life science research that, based on current understanding, can be reasonably anticipated to provide knowledge, information, products, or technologies that could be misapplied to pose a significant threat with broad potential consequences to public health and safety, agricultural crops and other plants, animals, the environment, materiel, or national security” (Casadevall et al., 2015). DURC presents unique biosecurity hazards when compared to less risky biological research. Policies developed in the U.S., mandate risk-benefit assessments, oversight mechanisms, transparency, and collaborative efforts between government entities and research institutions to ensure the responsible conduct of government-funded high-risk research. Unfortunately, government funding now accounts for less than half of life science funding (https://www.economicstrategygroup.org/publication/seven-recent-developments/). Therefore, a new paradigm is needed to capture non-government-funded DURC such as the construction of infectious horsepox virus, a virus in the *Orthopox* genus, which is related to smallpox virus, funded by a U. S. pharmaceutical company (Noyce et al., 2018).

In the aftermath of the 2001 anthrax attack (Jernigan et al., 2002), significant concern about dual-use research was raised in scientific and government circles following the publication of two papers. In the first, Jackson et al. (2001) introduced the gene for interleukin 4 (IL-4) into mousepox virus in an effort to produce a contraceptive vaccine to control the wild mouse population. Unexpectedly, the mice inoculated with the recombinant virus died even though they were genetically resistant to mousepox virus infection or had been immunized against it. Thus, instead of creating a contraceptive, they created a virus with enhanced pathogenicity. It was feared that the findings could be a blueprint to construct a more virulent smallpox virus that would evade the immunity established by vaccination. The second paper (Cello et al., 2002) described the synthesis of full-length poliovirus cDNA by assembling oligonucleotides of plus and minus strand polarity. The synthetic poliovirus cDNA was transcribed by RNA polymerase into viral RNA, which translated and replicated in a cell-free extract resulting in the *de novo* synthesis of infectious poliovirus. The media framed this work as a recipe for the synthesis of viruses for use as a biological weapon. Others argued that poliovirus was relatively simple and that the synthesis of more complex viruses like influenza or smallpox would be far more challenging. However, technology advances rapidly, and within 3 years, the influenza virus responsible for the 1918 pandemic, which resulted in an estimated 20 million deaths, was reconstructed using reverse genetics (Trumpy et al., 2005).

In 2005, a National Science Advisory Board for Biosecurity (NSABB) was established to provide advice, guidance, and leadership to the U. S. government regarding oversight of dual use life sciences research. Among its accomplishments, the NSABB developed a definition for DURC and identified seven specific categories of experiments that were likely to be worrisome (Imperiale and Casadevall, 2015). These are experiments that would: “1) Enhance the harmful consequences of a biological agent or toxin; 2) Disrupt immunity or the effectiveness of an immunization without clinical and/or agricultural justification; 3) Confer to a biological agent or toxin resistance to clinically and/or agriculturally useful prophylactic or therapeutic interventions against that agent or toxin or facilitates its ability to evade detection methods; 4) Increases the stability of, transmissibility of, or ability to disseminate a biological agent or toxin; 5) Alters the host range or tropism of a biological agent or toxin; 6) Enhances the susceptibility of a host population; and 7) Generates a novel pathogenic agent or toxin or reconstitutes an eradicated or extinct biological agent” (Casadevall et al., 2014a). These categories of experiments could help scientists, editors, and reviewers in deciding whether their proposed, ongoing work, or submitted manuscript is DURC.

In 2012, research groups in the Netherlands (Herfst et al., 2012), U.S. and Japan (Imai et al., 2012) produced mutated variants of the highly pathogenic H5N1 avian influenza virus that could infect ferrets via the airborne route. Though their stated aim was to determine what mutations could alter the host range of the virus, in order to be better prepared if such changes occurred in nature, the work provoked a heated debate in public and scientific circles, as to whether it should have been carried out at all, both for biosafety (the virus could escape the laboratory) and biosecurity (a blueprint for those with nefarious intent) reasons (Wain-Hobson, 2014; Nixdorf, 2024). This type of DURC has been termed gain-of-function (GOF) research because the modified H5N1 virus gained the ability to infect ferrets, which it did not have before (Casadevall et al., 2014b).

To address the biosecurity issues raised by these articles, some governments, academic institutions, professional societies, and journals subsequently developed policies concerning the publication of DURC. Science, Nature Publishing Group and the American Society for Microbiology journal group developed dual-use review policies (Resnick et al., 2011; Casadevall et al., 2015). However, results of a 2011 survey of 155 journals indicated that relatively few (N = 12) had a written dual-use policy and only nine said they had experience reviewing dual-use research in the past 5 years (Resnick et al., 2011). It is not known if the situation has improved over the ensuing years.

A more recent and problematic development for biosecurity is Open Science, which is a set of practices that aim to improve the reliability and efficiency of scientific research and are generally characterized by increased transparency (Smith and Sandbrink, 2022). Although, Open Science may encourage the exercise of best practices, there are also instances where it may also contribute to biosafety and biosecurity risk. Of particular importance to DURC, is the use of preprints, which are author-formatted articles publicly deposited in a repository. These preprints are a recent and novel way to disseminate microbiology research prior to formal peer review (Schloss, 2017). Preprints now account for 4% of all research articles (Yoshizawa et al., 2024). In one study, Malicki et al. (2020) found that only 68% of preprint servers provided some form of screening or moderation before the article was made public though not all of the screening involved dual-use, safety, or biosecurity-related criteria to mitigate the risk from publishing DURC.

## Frontier 3: synthetic biology

In 2017, the U. S. Department of Defense requested that the National Academies of Sciences, Engineering, and Medicine (NASEM) address the changing nature of the biodefense threat in the age of synthetic biology. The final report was published in 2018 (NASEM, 2018). Synthetic biology [now called engineering biology (Di Euliis et al., 2024)] is a branch of science within biotechnology that comprises a broad range of methodologies from various disciplines that enable the modification of biological organisms. These engineering biology approaches have the potential to be used in ways that could change the presentation of a bioterrorist attack. The final report (NASEM, 2018) stated that “engineering biology expands what is possible by creating new bioweapons and also expands the range of actors who could undertake such efforts and decreases the time required.” Three potential capabilities were considered to be most concerning: (1) re-creating known pathogenic viruses, (2) making existing bacteria more dangerous, and (3) making harmful biochemicals via *in situ* synthesis. With regard to biochemicals, engineering biology blurs the line between biological and chemical weapons. It can allow the delivery of biochemicals by a biological agent (Galanie et al., 2015). It may also be possible to modulate human physiology and behavior in novel ways by potentially allowing the engineering of microorganisms, the microbiome, or immune system (Borzenkov et al., 1994). More recently, the integration of AI with engineering biology has introduced biosecurity challenges that will need to be addressed (De Haro, 2024).

DNA synthesis technologies are catalyzing rapid advances in engineering biology. While the promise of this technology is immense, so is its potential for intentional or accidental misuse. Nucleic acids underpin much of research and development in the life sciences and serves as a critical control point. In 2010, in the interest of biosecurity, the U. S. Department of Health and Human Services (HHS) issued screening guidance for commercial providers of synthetic double-stranded DNA (dsDNA), which called on providers to voluntarily screen all orders (as cited by Diggans and Leproust, 2019). Subsequently, members of the International Gene Synthesis Consortium (IGSC), which represents about 80% of global gene synthesis capacity, implemented the Harmonized Screening Protocol (HSP). IGSC members screen every gene order against the DNA sequences in a common curated Regulated Pathogen Data Base, and against all entries found in internationally coordinated sequence databases (e.g., NCBI/GenBank) (IGSC, 2017).

In October 2023, Executive Order 14110 was issued and included a section, in response to reducing the risks of misuse of synthetic nucleic acids by improving associated biosecurity measures (Presidential Documents, 2023). In response, the new guidance expanded the definition of Sequences of Concern (SOCs) to include all sequences that contribute to pathogenicity or toxicity, whether from regulated (e.g., Select Agents) or unregulated agents. To ensure that synthetic nucleic acids are distributed and used responsibly, the guidance recommends that providers and third-party vendors verify the legitimacy of each recipient of nucleic acids containing SOCs, and that all parties maintain records of SOC transfers. The guidance also recommends that the manufacturers of benchtop nucleic acid synthesis equipment verify the legitimacy of their customers. The framework incorporates and supplements the 2023 HHS guidance (HHS, 2023).

## Conclusion

Advances in biotechnology and developments in artificial intelligence (AI) have resulted in a rapidly evolving threat landscape, which has presented new challenges for preventing the malicious, reckless, or accidental misuse of the life sciences.
